# Development and Validation of the Outcome Expectancy Scale for COVID-19 Vaccination in the Adult General Population

**DOI:** 10.3390/vaccines11010085

**Published:** 2022-12-30

**Authors:** Yanqiu Yu, Vivian W. I. Fong, Mason M. C. Lau, Joseph T. F. Lau

**Affiliations:** 1 Department of Preventive Medicine and Health Education, School of Public Health, Fudan University, Shanghai 200032, China; 2 Key Laboratory of Public Health Safety, Fudan University, Shanghai 200032, China; 3 Centre for Health Behaviours Research, Jockey Club School of Public Health and Primary Care, The Chinese University of Hong Kong, Hong Kong SAR, China; 4 Zhejiang Provincial Clinical Research Center for Mental Disorders, The Affiliated Wenzhou Kangning Hospital, Wenzhou Medical University, Wenzhou 325000, China; 5 School of Mental Health, Wenzhou Medical University, Wenzhou 325000, China; 6 School of Public Health, Zhejiang University, Hangzhou 310058, China

**Keywords:** COVID-19 vaccination, cognition, outcome expectancy, scale development, validation, China

## Abstract

Promotion of COVID-19 vaccination requires understanding its determinants, an important one of which is outcome expectancy. However, reliable and valid measurement tools are absent. This study thus developed and validated an Outcome Expectancy Scale for COVID-19 Vaccination (OES-COVID-19). An inductive approach was used for scale development; content validity of the items was then assessed by an independent evaluation expert panel. Iteratively, 17 scale items were created and validated in a population-based telephone survey conducted among 500 adults of the general population in Hong Kong, China, from August to September 2021. Using half of the sample (*n* = 249), exploratory factor analyses identified four factors of the OES-COVID-19, including (a) positive contribution to society, (b) functional benefits, (c) protective effect, and (d) negative impacts. Confirmatory factor analysis of these factors conducted on the second subsample (*n* = 251) was satisfactory. The OES-COVID-19 showed good psychometric properties in terms of internal consistency, absence of floor/ceiling effects, and concurrent validity. The newly created and validated OES-COVID-19 is deemed suitable for application. It may advance future studies related to COVID-19 vaccination behavior and intention, and potentially allows for comparisons across studies. Further validation with modifications across countries, populations, and phase of the pandemic are warranted.

## 1. Introduction

As of 14 October 2022, more than 12.7 billion doses of COVID-19 vaccines had been administered globally [1]. While a few countries (e.g., China and Canada) have achieved vaccination rates of at least 80%, many countries’ vaccination rates have stagnated (e.g., the U.K. and the U.S.) [1]. Pre- and post-rollout studies conducted in various countries have reported high levels of vaccination hesitancy [2,3,4,5,6,7,8] and vaccination resistance, i.e., many people indicated a clear choice of not going to take up COVID-19 vaccination [9,10]. As a high vaccination rate is required to control the pandemic, it is warranted to investigate the determinants of vaccination behaviour/intention/hesitancy.

Outcome expectancy refers to anticipated positive or negative outcomes resulting from performance of a particular behavior [11]. Positive and negative outcome expectancies are important determinants of COVID-19 vaccination, as health economists have pointed out that decisions are largely dependent on weighing potential gains and losses of a particular behavior [12]. The construct of outcome expectancy is multi-dimensional. Perceived efficacy and side effects represent key positive and negative expected outcomes of COVID-19 vaccination, respectively; such factors are among the most commonly reported cognitive factors of COVID-19 vaccination behavior and hesitancy [13]. Other potential positive outcomes of COVID-19 vaccination may involve psychological reliefs from worry about infection, which was associated with COVID-19 vaccination intention [14]. As more and more governments are opening their borders to vaccinated travelers while prohibiting unvaccinated people from accessing public venues (e.g., museums and restaurants) and attending activities (e.g., work/school/religious ceremonies) [15], people may perceive positive outcomes of COVID-19 vaccination in terms of functional advantages such as being waived from such restrictions. As COVID-19 vaccination can be seen as a prosocial behavior [16], some people may extend positive outcome expectancy to considering societal benefits such as herd immunity and recovery of the local economy [17]. Moreover, negative outcome expectancy such as insufficient protection effect is common [18]. Some of these perceptions on outcomes were associated with COVID-19 vaccination intention and behavior [13,14,17,18,19]. However, no study has systematically looked at the comprehensive range of positive/negative outcome expectancies perceived by the general public.

Numerous studies have investigated factors potentially related to outcome expectancy of COVID-19 vaccination (e.g., [8,18,19,20,21]). However, almost all of them constructed their own items or scales and did not examine their psychometric properties (e.g., factor structures and validity). Comparisons across studies is thus not feasible. While some scales have been developed to assess outcome expectancy of specific health-related behaviors (e.g., the Outcome Expectancy Scale for HIV serostatus disclosure [22] and the Anticipated Effects of Cannabis Scale [23]), there is nil regarding COVID-19 vaccination. A few scales were developed to look at cognitive factors other than outcome expectancy of COVID-19 vaccination, such as Perceptions of Causes of COVID-19 scale and the COVID-19 Perceived Risk Scale [24,25]. To our knowledge, only one of them (the three-item Impacts Subscale of the Drivers of COVID-19 Vaccination Acceptance Scale) looked at a single dimension of protection as the outcome expectancy of COVID-19 vaccination [26,27]; it was not developed specifically for COVID-19 vaccination but was modified from the Motors of Influenza Vaccination Acceptance Scale. While COVID-19 is dramatically different from influenza, that scale did not undergo a scale development process. Thus, to understand vaccination behavior and inform promotion, there is both a need and a gap to develop validated scales to assess outcome expectancy of COVID-19 vaccination in a comprehensive manner. The Outcome Expectancy Scale for COVID-19 vaccination (OES-COVID-19) was thus developed in the present study.

The development of the OES-COVID-19 may also facilitate theory development, as outcome expectancy is an important construct of a number of commonly used behavioral health theories, including the Health Action Process Approach (HAPA) model [28] and the Social Cognitive Theory (SCT) [11]. The HAPA model proposes that outcome expectancy is chiefly important in the intention phase prior to performing a behavior, during which an individual balances the pros and cons of the potential consequences of the behavior of interest [28]. Favorable outcome expectancy would motivate an individual to initiate or maintain the behavior [29]. The SCT postulates that individuals’ behavior is determined by reciprocal determinism that involves interactions among the environment, individual characteristics, and behavior. Its key constructs include outcome expectancy, self-efficacy, observational learning, and reinforcement [11]. Both theories have been applied to understand a wide range of health-related behaviors, including influenza vaccination [30,31] and COVID-19 vaccination intention [21].

Given the background, this study developed and validated the OES-COVID-19 in a sample of Chinese adults of the general population in Hong Kong. We investigated its psychometric properties including factor structure, floor and ceiling effects, internal consistency, and concurrent validity. Two external variables were selected to assess concurrent validity. The first one was performed or scheduled COVID-19 vaccination as outcome expectancy of COVID-19 vaccination is a known predictor of vaccination intention/behavior [8,18,30,31]. The second one was attitude towards overall benefits versus cost of COVID-19 vaccination as the outcome expectancy of a particular behavior was associated with such attitudes [32].

## 2. Materials and Methods

### 2.1. Scale Development of the OES-COVID-19

#### 2.1.1. Item Generation and Selection

The item generation process was guided by previous key literature on scale development [33,34,35]. An inductive approach was deployed to generate the items: (1) Semi-structured interviews were conducted in a purposively selected sample of 24 Hong Kong Chinese adults; 67% of whom were female; they were evenly distributed in the 18–35, 36–50, and 51–75 age groups. Phone interviews were conducted because of the social gathering restrictions during the COVID-19 pandemic. Interviews rather than focus groups were conducted as the literature reported that the former would result in better validity than the latter [36]. (2) The interviewees were requested to list as much as possible their perceived potential positive and negative outcomes of COVID-19 vaccination; prompts were given during pauses. A total of 127 initial items were hence generated during these interviews.

The item selection process was then performed by an expert scale development panel, with three members having research background of infectious diseases, psychology, public health, and behavioral medicine. The panels coded, simplified, and collated the collected items. Items with similar meanings were grouped together; items unrelated to outcome expectancy (e.g., “I don’t want to try a new vaccine”) and very rarely mentioned (e.g., “taking up COVID-19 vaccination or making an appointment to do so would spend too much time”) were removed from the list. A pool of 15 initial items (10 on positive outcome expectancy and five on negative outcome expectancy) were hence created for further evaluation. They were transformed into 15 question items being rated on five-point Likert scales (1 = Strongly disagree to 5 = Strongly agree).

#### 2.1.2. Establishment of Content Validity

Content validity was established following methods recommended in previous scale development studies [33,34,35]. An independent evaluation panel was formed, which comprised seven experts having 10 to 25 years of extensive research experience in public health, health psychology, and behavioral medicine, including five local members and two non-local members. The panel reviewed the 15 proposed items written in Chinese, and rated each item in terms of its relevance, clarity, and importance regarding the construct of interest, using four-point Likert rating scales (1 = not relevance/clear/important to 4 = highly relevant/clear/important). The scores of the Content Validity Index were then calculated at both the item level (i.e., I-CVI) and the scale level (i.e., S-CVI), according to the three afore-mentioned dimensions of content validity (i.e., relevance, clarity, and importance). The I-CVI values were the proportion of experts of the evaluation panel scoring 3 or 4; values of <0.50, 0.50–0.78, and >0.78 would indicate rejection of the item, acceptable content validity, and good content validity, respectively [37,38]. The S-CVI was derived by dividing the number of items scored 3 or 4 by the product of the number of experts (seven) and the number of scale items (15); S-CVI of >0.80 and >0.90 would indicate acceptable and excellent content validity, respectively [37]. Members of the evaluation panel were further asked to provide feedbacks on whether the items had covered all important aspects of outcome expectancy of COVID-19 vaccination, and to recommend any additional potentially important items. Based on the two indices and recommendations, the scale development expert panel revised the items. The revised version was then sent back to the independent evaluation panel to repeat the rating exercise. The scale development expert panel then met again to discuss the revised ratings and comments and finalized the items through a consensus procedure. Data of the scale development processes are presented in the Results Section.

### 2.2. The Validation Survey: Participants and Data Collection

To validate the OES-COVID-19 scale developed by the aforementioned procedures, a random telephone survey was conducted in a Chinese population aged ≥18 years in Hong Kong, China from 4 August to 1 September 2021. All participants were Chinese speakers. It was administered between 5 to 10 pm to avoid over-sampling non-working individuals. Telephone numbers were randomly drawn from the most updated residential telephone directory. Unanswered telephone calls were given at least three attempts before defining the telephone number as an invalid one. The eligible household member whose birthday was closest to the survey date was interviewed. Appointments were made if necessary. Trained interviewers briefed the participants about the study, sought their verbal informed consent, and signed a form pledging completion of the required consent procedures. No incentives were given to the participants. Participants could quit at any time without being questioned. The response rate, i.e., the number of completed interviews (500) divided by the number of eligible contacts (897), was 55.7%. Ethics approval was obtained from the corresponding author’s affiliated institution (Ref No. SBRE-20-850).

### 2.3. Other Measures Used in the Validation Survey 

Background information was collected, including sex, age, educational level, marital status, and whether suffering from one of the listed chronic diseases (e.g., hypertension, diabetes, and chronic pulmonary diseases). Two external variables were used to establish concurrent validity: (1) Attitude towards overall benefits versus cost of COVID-19 vaccination was assessed by the level of agreement with the statement “Overall, the benefits of taking up COVID-19 vaccination outweigh its costs (1 = strongly disagree to 5 = strongly agree)”; (2) Performed or scheduled COVID-19 vaccination was assessed by an item asking whether the participants had taken up at least one dose of COVID-19 vaccination or made an appointment to do so (yes/no).

### 2.4. Data Analysis of the Validation Study 

Following the recommendation and practice of previous literature [39,40,41], the overall sample was randomly split into two nearly even halves. Exploratory factor analysis (EFA) was conducted in one subsample (*n* = 249), and confirmatory factor analysis (CFA) was conducted in another subsample (*n* = 251). Regarding the EFA, the common factor analysis (Principle Axis Factoring) and oblique rotation (the Promax method) were performed to extract factors from the OES-COVID-19. Items with double/multiple loadings (i.e., those with factor loadings >0.4 on multiple factors) and those with all factor loadings <0.4 were removed. EFA was performed again after removal of each item; the process was iterated until no item needed to be removed further. The number of factors to be extracted was determined by the scree plot and the conventional Kaiser criteria (i.e., eigenvalue > 1.0). In the second subsample, CFA using the maximum likelihood estimation method was conducted to confirm the factor structure identified by EFA. Goodness-of-fit statistics and cut-off criteria for CFA included χ2/df < 5.00, Comparative Fit Index (CFI) > 0.90, Tucker-Lewis Index (TLI), and Root Mean Square Error of Approximation (RMSEA) < 0.08. In addition, internal consistency was assessed by Cronbach’s alpha in the entire sample. The OES-COVID-19 would be considered as having a floor effect or a ceiling effect if more than 15% of the participants scored the minimum score or maximum score on any of the identified factors of the OES-COVID-19. Concurrent validity was assessed by deriving Pearson and Spearman correlation coefficients between each of the identified factors and each of the two external variables (i.e., attitude towards overall benefits versus cost of COVID-19 vaccination and performed or scheduled COVID-19 vaccination). CFA was conducted by using AMOS 26.0 while the other tests were analyzed by SPSS 23.0. Statistical significance was defined as *p* < 0.05 (two-tailed tests).

## 3. Results

### 3.1. Content Validity of the OES-COVID-19

In the first-round assessment conducted by the independent evaluation panel, 93.3% of the items showed an I-CVI of ≥0.78 regarding relevance (ranged from 0.71 to 1.00) and importance (ranged from 0.71 to 1.00), but the proportion of items having I-CVI ≥ 0.78 regarding clarity was only 33.3% (a range of 0.57 to 0.86). Similarly, the S-CVI indicated excellent content validity in terms of relevance and importance (0.92 and 0.94, respectively), but not clarity (S-CVI = 0.73). Based on such results, the items’ wordings were refined by the first scale development panel to improve clarity. In addition, two new items recommended by the independent panel were added to the item pool, which then had 14 items on positive outcome expectancy and four on negative outcome expectancy. The 15 revised items and the two newly created items were resent to the independent panel for rating. The results of this second round of content validity evaluation showed that the 17-item scale were all acceptable in terms of relevance, clarity, and importance at both the item-level (I-CVI ranged from 0.71 to 1.00) and the scale level (S-CVI ranged from 0.92 to 0.97). Good content validity had thus been demonstrated. The 17-item OES-COVID-19 was slightly revised by the first panel and then used in the validation survey. 

### 3.2. Characteristics of the Participants of the Validation Survey

The results are shown in Table 1. Of all the participants, over half were females (67.0%), aged 36–65 (52.0%), having attained an educational level of middle school or below (56.8%), and being currently married (64.6%); 28.2% self-reported having at least one of the listed chronic diseases. Regarding COVID-19 vaccination behavior, 61.0% had taken up at least one dose of COVID-19 vaccination or made an appointment to do so. The mean score of the attitude towards overall benefits versus cost of COVID-19 vaccination was 3.5 (standard deviation (SD): 0.8; range: 1–5) a higher score implies a more favorable attitude (i.e., benefits outweighed cost).

### 3.3. EFA

The Kaiser-Meyer-Olkin Measure of Sampling Adequacy (KMO = 0.84) and the Bartlett’s Test of Sphericity (Chi-square (*df* = 136) = 4082.0; *p* < 0.001) yielded satisfactory results, indicating that the subsample (*n* = 249) was suitable for EFA. In EFA, one item (“Your taking up COVID-19 vaccination would reduce the chance of death in case of COVID-19 infection”) of the initial 17-item OES-COVID-19 was removed due to double factor loadings. Another item was removed because all its factor loadings were lower than 0.4 (“Your taking up COVID-19 vaccination would reduce the chance of severe disease symptoms in case of COVID-19 infection”).

The final version of EFA applied to the remaining 15 items yielded four factors according to the scree plot and the criterion of eigenvalue >1. The results are summarized in Table 2. (1) The first four-item factor was “Positive Contribution to Society”; the items were related to perceived societal benefits of COVID-19 vaccination such as economic recovery and relief of travel restrictions. (2) The second five-item factor was Functional Benefits; the items were related to relaxation or removal from restrictions of preventive measures in daily life and psychological relief from COVID-19 infection. (3) The third three-item factor was Protective Effect; the items were related to the vaccine’s protection of oneself, family members, and others from contracting COVID-19. (4) The final three-item factor was “Negative Impacts”; the items were related to potential negative impacts of COVID-19 vaccination concerning physical harm (e.g., mild or severe side effects) and interruptions of daily life/work. These four factors showed eigenvalues of 6.9, 2.2, 1.3, and 1.2, and had explained 45.7%, 14.9%, 8.7%, and 8.2% of the total variance (77.5%), respectively.

### 3.4. CFA

Using another half-split subsample, the CFA was applied to the four-factor structure identified in the EFA. The results are shown in Table 2, which demonstrated satisfactory goodness-of-fit, i.e., χ^2^/*df* = 251.66/84 = 3.00, CFI = 0.96, TLI = 0.95, and RMSEA = 0.90. The standardized path estimates ranged from 0.39 to 0.99 (all *p* < 0.001). The factor of negative impacts was negatively associated with the factor of positive contribution to society (*r* = −0.32; *p* < 0.001), but not with that of protective effect (*r* = −0.02; *p* = 0.809), nor with functional benefits (*r* = −0.01; *p* = 0.964). The factors of positive contribution to society functional benefits, and protective effect were positively correlated with each other (*r* ranged from 0.59 to 0.79; all *p* < 0.001).

### 3.5. Floor and Ceiling Effect

The mean (SD; range) scores were 10.3 (2.2; 3–15) for the factor of positive contribution to society, 16.7 (4.1; 5–25) for the factor of functional benefits, 13.6 (2.7; 4–20) for the factor of protective effect, and 9.9 (2.0; 3–15) for the factor of negative impacts. Neither floor nor ceiling effects were observed as all the proportions of minimum scores (a range of 0.2% to 0.4%) and maximum scores (a range of 1.2% to 3.4%) of the four factors were much lower than 15% (see Table 3).

### 3.6. Reliability and Validity

The internal consistency of all the four factors of the OES-COVID-19 was acceptable, with Cronbach’s alpha ranging from 0.7 to 0.9 (see Table 3). Regarding concurrent validity, the three factors of positive outcome expectancies (positive contribution to society (r = 0.51, *p* < 0.001), functional benefits (*r* = 0.59, *p* < 0.001), and protective effect (*r* = 0.58, *p* < 0.001)) were all positively correlated with attitude towards overall benefits versus cost of COVID-19 vaccination, which was negatively correlated with the factor of negative impacts (*r* = −0.10, *p* = 0.026). Similarly, the three factors of positive outcome expectancy were all positively correlated with performed or scheduled COVID-19 vaccination (*r* = 0.36, 0.34, and 0.39, respectively; all *p* < 0.001), which was negatively correlated with the factor of negative impacts (*r* = −0.24, *p* < 0.001).

## 4. Discussion

To our knowledge, the OES-COVID-19 is the only tool assessing specific outcome expectancies of COVID-19 vaccination. The items were generated inductively. They included a comprehensive set of positive and negative expected outcomes of COVID-19 vaccination that impact various aspects of daily life. Its development followed rigorous iterating scale development and evaluation processes conducted by two independent expert panels. Content validity in terms of clarity, relevance, and importance was satisfactory. Its factor structure was examined in two half-split subsamples. The EFA applied to the first subsample generated four highly interpretable factors of positive contribution to society, functional benefits, protective effect, and negative impacts. These four constructs were confirmed in the second subsample using CFA. Internal consistency of the four identified factors was satisfactory. Notably, the Cronbach’s alpha of Factor 4 of Negative Impacts was acceptable but relatively low (0.7), probably because two items are related to the side effects and one related to the impacts on daily life. In addition, neither ceiling effect nor floor effect of the OES-COVID-19 was observed. The scale factors were associated with vaccination behavior and attitude toward overall benefits versus cost of COVID-19 vaccination, demonstrating concurrent validity. Thus, the OES-COVID-19 developed in the present study has been carefully validated and is ready to use. It can potentially contribute to understanding and predicting COVID-19 vaccination behavior, vaccine hesitancy, and vaccine resistance. 

The first factor of Positive Contribution to Society factor explained the largest percentage of variance (45.7%) among the four identified factors. In the present study, this factor was significantly associated with vaccination behavior. The findings have an important implication that COVID-19 vaccination, like influenza vaccination, can be seen as a prosocial behavior [16]. A number of studies found that prosociality and collectivism were positively associated with COVID-19 vaccination intention among Chinese university students [42,43]. In addition, a study reported that the perceived contribution to controlling COVID-19 as a result of a high vaccination rate was positively associated COVID-19 vaccination intention among Chinese healthcare workers [14]. Thus, perceived positive outcomes are not limited to self-interest but may also include perceived societal benefits. 

The second factor is related to the functional benefits of COVID-19 vaccination. Doubtlessly, the strict measures implemented to control the pandemic, such as lockdown, quarantine, social distancing, working from home, and travel restrictions, have interrupted many aspects of normal life [44,45]. Many governments (e.g., Australia and the U.K.) have implemented mandatory vaccination in special functional groups (e.g., healthcare professionals, teachers and university students, nursing home workers, and restaurant staff) [15]; vaccination status has thus increasingly affected work and daily life. Some governments (e.g., the U.K and Thailand) are opening their borders only to vaccinated travelers and the concept of ‘travel visa’ has been widely discussed. In a number of countries, some public venues such as night clubs, bars, museums, and churches are also only open to vaccinated people [46,47]. In many countries, vaccination has thus become a prerequisite for returning to a more ‘normal’ life. Hence, COVID-19 vaccination may result in functional benefits alleviating or waiving related restrictions. Furthermore, literature has consistently shown that these control measures and worry about infection (for oneself and family) were associated with mental distress, such as depression, anxiety, acute stress, and post-traumatic stress symptoms in the general and special populations [48,49,50,51]. Notably, one of the potential functional benefits of vaccination is to reduce such worries.

The third factor is protective effect, which is expected as the literature has commonly reported significant associations between perceived protective effect resulted from vaccination and vaccination intention/behaviors [13,21]. Indeed, most of previous studies focused on this construct of efficacy of the vaccines or vaccines’ ability in protecting people from infection when considering perceived benefits of COVID-19 vaccination [8,18,19,20,21]. In the present study, protection was not the most important construct of the scale in terms of percentage of variance explained. This is understandable in the context as Hong Kong only had a low number of new COVID-19 infection reported (only 119 cases during the entire survey period).

The fourth factor of Negative Impacts (e.g., side effect) is also expected. Positive and negative outcomes are two different sides of the decision balance. Negative impacts was not significantly associated with or only mildly associated with the three other factors of positive outcome expectancies. Protection and functional benefits might occur independently in the presence of prevalent side effects. Hence, both aspects should be considered in interventions promoting COVID-19 vaccination. The three types of positive outcomes were positively associated with each other, which is also understandable as they may have common roots. For instance, high efficacy of COVID-19 vaccines and a high population vaccination rate may result in all three types of positive outcomes; some societal outcomes (e.g., herd immunity) may also result in functional benefits at the individual level.

The validated scale shows satisfactory concurrent validity, as demonstrated by the significant associations between its four subscales and COVID-19 vaccination intention/behavior. The validated scale has multiple beneficial public health implications. Eradication of COVID-19 is unlikely; boosters or annual COVID-19 vaccination may become necessary. Vaccine hesitancy is dynamic and might soar again due to changes in perception and the ‘returning to normal life’ after lifting the control measures. To update related health promotion, serial surveillance of changes in perceived outcomes of COVID-19 vaccination is warranted. Importantly, such health promotion may consider modifying the levels of the four identified constructs of the OES-COVID-19 to increase motivations of COVID-19 vaccination, i.e., to increase the positive outcome expectancies and to reduce the negative outcome expectancies. Previous work mainly focused on the benefits of protection. However, apart from the construct of protection, the scale’s two other significant positive outcome expectancies (societal and functional benefits) have been underemphasized; assessment of the three dimensions of positive outcome expectancies widens the horizon of understanding the positive consequences of COVID-19 vaccination. Hence, health promotion should emphasize the positive societal outcomes of COVID-19 vaccination, such as its potential positive impacts on herd immunity and recovery of the economy. Citizens in countries preparing to remove control measures should be explained that high vaccination rates would not only protect oneself and others, but also be an important prerequisite of minimizing COVID-19 deaths and speeding up removal of the control measures. As many countries have linked the key COVID-19 control measures to various aspects of daily life or started removing these measures, it seems that the level of functional benefits of COVID-19 vaccination may change over time. The application of the Functional Benefits subscale of the OES-COVID-19 may help monitor changes in people’s responses to the changing COVID-19 policies and understand the changes in the associations between functional benefits and vaccination intention/behaviors at different stages of the pandemic. The presence of the Negative Impacts subscale reminds us that it is essential to convince the public about the safety of COVID-19 vaccines, especially for older people with controlled chronic disease conditions, as unvaccinated older people are exposed to higher risk of COVID-19 deaths [52,53]. As the levels of the four factors were only moderate among adults of the Hong Kong general population, there are still substantial rooms for improvement. If predictive validity is confirmed in future longitudinal studies, interventions may be designed to change these four modifiable dimensions of outcome expectancies of COVID-19 vaccination.

The new scale may also contribute to testing behavioral theories (e.g., HAPA and SCT) in the context of COVID-19 vaccination. For instance, the widely applied Theory of Planned Behaviors (TPB) postulates that attitude, subjective norm, and perceived behavioral control determine behavioral intention, which further determines behavior [54]. It postulates that formation of attitude is based on considering the likelihood of specific expected outcomes (i.e., outcome expectancy) weighted by perceived desirability of such outcomes (i.e., values) [32]. The postulation was supported in the present study, as the four factors of the OES-COVID-19 were significantly associated with attitude towards overall benefits versus cost of COVID-19 vaccination. Thus, modification of outcome expectancy might facilitate the formation of positive attitude towards COVID-19 vaccination, which is a known predictor of vaccination intention [55].

It is necessary to recognize variations in the levels and importance of the scale’s constructs in affecting vaccination decision. The COVID-19 pandemic is dynamic in nature. The benefits of COVID-19 vaccination might change across the stages of the pandemic. For instance, the levels of the protection and negative impacts might differ in societies according to the epidemiology of the pandemic (e.g., severity and the number related to deaths); the levels of perceived societal and functional benefits may also depend on the socio-cultural contexts. Despite such potential variations, this new scale has good potentials for comparisons across countries and stages of the pandemic as the subscales include general determinants of vaccination decisions (e.g., protection [13], societal benefits [17], functional benefits [15], and negative impacts [13]). The items were also generated through an inductive approach. It is likely that the levels of constructs would vary according to the context, but it is also likely that the factor structure and other psychometric properties would be stable across countries. Cross-cultural validations and comparisons are warranted for confirmation. As reducing vaccine hesitancy against COVID-19 vaccination has long-term significance, this study is a good starting point to develop a scale assessing major determinants of COVID-19 vaccination. Future validations may modify or add some items if necessary.

The present study has several limitations. First, this study did not examine test–retest reliability. Second, only two concurrent validity measures were tested, while a single item was used to measure general attitudes. Third, although the response rate was comparable to other local telephone surveys [56,57], characteristics between the respondents and non-respondents might differ. While the age distribution of population in this study was comparable to that of the 2019 Hong Kong census data, the proportion of females was slightly over-represented. Lastly, social desirability bias may exist, as COVID-19 vaccination was a socially desirable behavior. Participants might tend to report perceptions favorable to COVID-19 vaccination.

In conclusion, this study developed and validated the OES-COVID-19, which consisted of four factors (i.e., positive contribution to society, functional benefits, protective effect, and negative impacts). It showed satisfactory psychometric properties and is deemed suitable for application. The availability of the validated scale can potentially advance research on COVID-19 vaccination. Future studies are needed to test the factor structure across time, countries, and populations. Modifications are possible. Longitudinal studies looking at its predictive validity and randomized controlled trials investigating the efficacy of modifying its constructs are greatly warranted. In surveillance studies, the scale can be used to track changes in peoples’ beliefs about the perceived positive and negative consequences of COVID-19 vaccination. In addition, the scale’s constructs can be used to design vaccine promotion interventions and test mediation mechanisms of the efficacy of such interventions. The scale may also facilitate future research about the development of behavioral health theories on vaccination behaviors and testing empirically the impacts of outcome expectancy on vaccination outcomes. The validated OES-COVID-19 is a reliable, valid, and potentially useful tool for advancing COVID-19 vaccination research.

## Figures and Tables

**Table 1:** Characteristics of the participants of the validation survey (*n* = 500).

	*n*	%
Sex		
Female	335	67.0
Male	165	33.0
Age groups (years)		
18–35	118	23.6
36–65	260	52.0
>66	121	24.2
Refused to answer	1	0.2
Educational level		
Primary school or below	119	23.8
Junior/senior middle school	210	42.0
College or above	161	32.2
Refused to answer	10	2.0
Marital status		
Others	173	35.6
Married	323	64.6
Refused to answer	4	0.8
Chronic disease status		
No/Don’t know	357	71.4
Yes	141	28.2
Refused to answer	2	0.4
Performed or scheduled COVID-19 vaccination		
No	195	39.0
Yes	305	61.0

Notes: Chronic disease status was defined as those who self-reported having one of the listed chronic diseases, including hypertension, diabetes, chronic pulmonary disease, myocardial infarction, heart failure, cerebrovascular disease, Alzheimer’s disease, ulcerative diseases, hepatic diseases, and tumors.

**Table 2:** Exploratory and confirmatory factor analysis on the OES-COVID-19 using half-split subsamples.

	Exploratory Factor Analysis (*n* = 249) (Factor Loading)				Confirmatory Factor Analysis (*n* = 251) (Path Estimate)
	Factor 1	Factor 2	Factor 3	Factor 4	
**Factor 1: Positive contribution to Society**					
Item 1. Your taking up COVID-19 vaccination would contribute to improving local economy and employment situation in Hong Kong.	**0.89**	0.03	0.04	0.03	0.94 ***
Item 2. Your taking up COVID-19 vaccination would contribute to loosening of the oversea travel restrictions on Hong Kong people.	**0.91**	−0.02	−0.01	0.01	0.93 ***
Item 3. Your taking up COVID-19 vaccination would contribute to reducing the overall negative impacts of the COVID-19 pandemic on Hong Kong.	**0.92**	−0.02	−0.00	0.02	0.96 ***
Item 4. Your taking up COVID-19 vaccination would contribute to achieving herd immunity and preventing transmissions of COVID-19 in the community.	**0.84**	0.03	−0.01	−0.02	0.86 ***
**Factor 2: Functional benefits**					
Item 5. Your taking up COVID-19 vaccination would satisfy the anti-pandemic policy’s requirement and make your life more convenient (e.g., going to restaurants or particular venues).	−0.07	**0.95**	0.00	0.00	0.87 ***
Item 6. Your taking up COVID-19 vaccination would allow you feel free to join friends’ gatherings.	−0.09	**0.94**	0.01	−0.03	0.86 ***
Item 7. Your taking up COVID-19 vaccination would facilitate your traveling overseas.	0.14	**0.65**	−0.16	−0.05	0.71 ***
Item 8. Your taking up COVID-19 vaccination would make you feel good.	0.09	**0.55**	0.19	0.03	0.83 ***
Item 9. Your taking up COVID-19 vaccination would reduce your worries about contracting COVID-19.	0.14	**0.53**	0.16	0.09	0.81 ***
**Factor 3: Protective effect**					
Item 10. Your taking up COVID-19 vaccination would prevent yourself from contracting COVID-19 (including the mutated variants) effectively.	−0.05	0.02	**0.99**	−0.05	0.99 ***
Item 11. Your taking up COVID-19 vaccination would prevent your family members from contracting COVID-19 (including the mutated virus) effectively.	0.00	−0.03	**1.00**	−0.00	0.99 ***
Item 12. Your taking up COVID-19 vaccination would prevent people other than your family members from contracting COVID-19 (including mutated variants) effectively.	0.06	0.00	**0.93**	0.02	0.97 ***
**Factor 4: Negative impacts**					
Item 13. It is possible that you may experience mild side effects (e.g., fatigue, headache, and fever) after taking up COVID-19 vaccination.	0.06	0.08	−0.01	**0.46**	0.39 ***
Item 14. It is possible that you may experience severe side effects and even death after taking up COVID-19 vaccination.	0.06	−0.10	0.00	**0.70**	0.63 ***
Item 15. It is possible that your daily life or work may be interrupted because of side effects after taking up COVID-19 vaccination.	−0.11	0.02	−0.03	**0.74**	0.79 ***
**Eigenvalue**	6.85	2.24	1.30	1.23	-
**Proportion explained**	45.68%	14.92%	8.67%	8.21%	-

Note: *** *p* < 0.001.

**Table 3:** Psychometric properties of the factors of the 15-item Outcome Expectancy Scale for COVID-19 Vaccination (*n* = 500).

	Range	Mean, SD	Proportion of the Minimum Score (%)	Proportion of the Maximum Score (%)	Internal Consistency (α)
Factor 1: Positive contribution to society	3–15	10.3, 2.2	0.2	3.4	0.9
Factor 2: Functional benefits	5–25	16.7, 4.1	0.2	2.6	0.9
Factor 3: Protective effect	4–20	13.6, 2.7	0.4	3.0	0.9
Factor 4: Negative impacts	3–15	9.9, 2.0	0.2	1.2	0.7

Note: SD = Standard deviation.

## Data Availability

Data is available upon reasonable request.
